# The brain uses extrasomatic information to estimate limb displacement

**DOI:** 10.1098/rspb.2015.1661

**Published:** 2015-09-07

**Authors:** Alexander V. Terekhov, Vincent Hayward

**Affiliations:** 1 University of Paris Descartes, Paris 05, UMR 8158, LPP, Paris 75006, France; 2 Sorbonne Universités, UPMC Univ Paris 06, UMR 7222, ISIR, Paris 75005, France

**Keywords:** perception, illusion, proprioception, touch

## Abstract

A fundamental problem faced by the brain is to estimate whether a touched object is rigidly attached to a ground reference or is movable. A simple solution to this problem would be for the brain to test whether pushing on the object with a limb is accompanied by limb displacement. The mere act of pushing excites large populations of mechanoreceptors, generating a sensory response that is only weakly sensitive to limb displacement if the movements are small, and thus can hardly be used to determine the mobility of the object. In the mechanical world, displacement or deformation of objects frequently co-occurs with microscopic fluctuations associated with the frictional sliding of surfaces in contact or with micro-failures inside an object. In this study, we provide compelling evidence that the brain relies on these microscopic mechanical events to estimate the displacement of the limb in contact with an object, and hence the mobility of the touched object. We show that when pressing with a finger on a stiff surface, fluctuations that resemble the mechanical response of granular solids provoke a sensation of limb displacement. Our findings suggest that when acting on an external object, prior knowledge about the sensory consequences of interacting with the object contributes to proprioception.

## 1. Introduction

When pushing on a concrete wall, one typically experiences the sensation that the wall is immobile. The mere act of pushing on a stiff surface nevertheless triggers the response of almost every type of mechanoreceptor in the body. Muscles contract, causing muscle spindles to fire [1], tendons become taut triggering the response of Golgi organs [2], and cutaneous receptors in the hand [3] and the hairy skin [4] become excited in great numbers. From this massive influx of somatic information, the brain is able to decide that, indeed, the wall and the hand in contact with it are immobile. Now, when pushing on a flexible tree, the same set of receptors provides sensory inputs not greatly different from that obtained when pushing on a concrete pole. Nevertheless, the hand and the tree are both felt to be moving. How could the solution of this effortless perceptual task come about?

During touch, limbs displace and deform objects, and their movements give rise to microscopic mechanical events arising from friction and micro-fractures in their bulk. These microscopic events can be sensed [5], and, given the mechanical properties of the object, can be used to determine the change of the object's mechanical state, which is indissolubly related to the displacement of the limb acting on it. We term such information extrasomatic information because it relates to objects that are external to the body.

The contributions of such prior exogenous information to perception have frequently been documented. For example, a prior assumption of the stationarity of the world underlies the interpretation of visual information [6,7]; the properties of the Doppler effect influence auditory perception [8], and so do learned relationships between pitch and movement [9]; the relationship between surface reaction forces and surface shape is used in shape perception through touch [10]; and the relation between length and moment of inertia of a wielded object influences distance perception in probe-mediated haptic exploration [11]. Moreover, similar principles may underlie the integration of intramodal sensory cues in different modalities [12]. It is thus likely that the processing of proprioceptive information could also benefit from prior knowledge about the laws of mechanical interaction. The influence of microscopic mechanics on the perception of bulk elasticity during active touch was recently reported [13,14], suggesting that this information is taken into account by the brain and can potentially contribute to the estimation of limb movements.

To test the hypothesis that the brain uses prior knowledge of the properties of a touched object to estimate limb movement, we asked observers to press on a stiff surface that produced microscopic fluctuations resembling the response of granular solids, the most common type of solid material on earth [15]. The fingertip stimulus was delivered to the skin through a dense array of small, millimetre-scale traction surfaces. The laws of contact mechanics determine how the patterns of fluctuations are distributed over the surface of a fingertip [16]. In the common case of interaction with a solid, the entire surface of contact oscillates uniformly, because the wavelength of the waves propagating in it is normally much greater than the size of a finger contact. For example, in a piece of wood, the celerity of waves is about 4000 m s^−1^, which suggests that at the upper limit of the tactile sensitivity (1 kHz), the wavelength is 4 m. In solids made of aggregate materials such as soils, the celerity of waves is an order of magnitude slower [17], but such solids still appear to a fingertip to oscillate as a single block. More complex patterns of oscillations in solids with wavelengths smaller than a finger contact are also possible, but their high frequencies and short decay periods place them far outside the range of the tactile sensitivity unless extremely particular conditions are arranged [18]. These acoustic considerations guided us to design two similar stimulation conditions that differed only by their spatial pattern. In the ‘in-phase’ condition, all traction surfaces moved approximately as a single body. In the ‘anti-phase’ condition, every neighbouring pair of traction surfaces moved in the opposite direction, creating a highly unusual oscillation pattern. We conjectured that if the brain indeed relies on the prior experience of interaction with touched objects, then an ‘ecological’ in-phase stimulation should elicit a sensation of finger displacement, whereas an atypical anti-phase stimulation should have little or no effect. The design of these stimuli is also supported by recent findings about the early stages of tactile input processing [19,20]. These studies respectively suggest that first- and second-order neurons selectively respond to classes of spatio-temporal input patterns present in natural stimuli, thus likely to transmit very different signals to the higher brain regions in the two conditions.

Three experiments comprising main and control conditions were conducted. In the main condition of experiment 1, all observers reported a strong illusory sensation of finger movement when, in fact, the surface admitted no macroscopic displacement, suggesting that the elaboration of a percept of limb position by the brain may use information extracted from its interaction with external objects. Experiment 2 demonstrated the central role played by congruency between the cutaneous input experienced by the observers and the pressing force that they produced. Finally, the results of experiment 3 showed that if the stimulus was delivered in combination with actual finger movement, the perceived finger displacement was a combination of the real and illusory displacements.

## 2. Material and methods

### (a) Observers

In experiment 1, 10 observers (six male, four female, with ages ranging from 23 to 30 years, with a mean of 28 years) volunteered to participate the study. They were naive to the experimental hypotheses, and had not taken part in any previous similar studies. In experiment 2 comprising a single control condition, three male observers (age range: 29–33 years) volunteered to take part in the testing. Ten new observers volunteered to participate in experiment 3 (five male, five female, with ages ranging from 23 to 36 years).

Observers reported no motor, sensory or neural disorders that could affect the outcome of the study.

### (b) Apparatus

**Figure 1.**
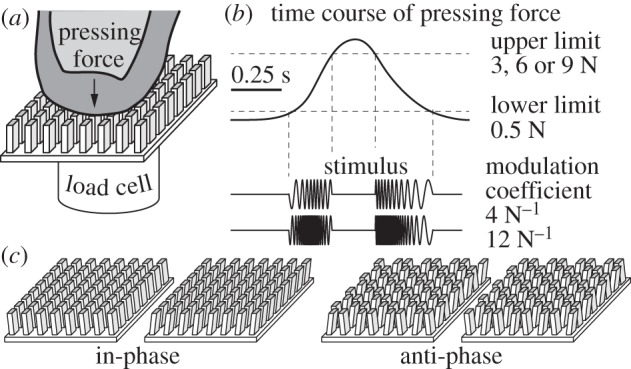
Stimuli description. (*a*) Observers pressed on an active surface comprising individually controlled, laterally moving, actuated elements. A load cell reported the vertical component of the applied force. A stiff servomechanism could optionally move the tactile active surface vertically. (*b*) The elements oscillated at a rate proportional to the temporal change of the pressing force. The oscillations occurred only when the force was above a constant lower limit and below a variable upper limit, at which point the observers stopped pressing. (*c*) In the in-phase condition, all elements moved in the same direction. In the anti-phase condition, each element moved in the direction opposite to that of its neighbours.

A distributed tactile stimulator (Latero, TactileLabs) was rigidly mounted onto a six-axis force sensor (FTD-NANO17, ATI) which acted as a load-cell, because only the vertical component of the measured interaction force was used. The stimulator had an active surface made of an 8 × 8 grid of contact laterally moving elements with a spatial period of 1.2 mm along the two directions. The entire surface of the grid was about 1 cm^2^. Each contact element could be individually commanded to move sideways by assigning a reference position, *p_ij_*, entraining the skin by traction without slip. Each individual traction surface had a size of 1.0 × 0.25 mm. The system is described elsewhere in detail [21], and the apparatus is schematically depicted by figure 1*a*. The force sensor was mounted on a vertically moving motorized platform based on a lab-jack mechanism (model L490/M, Thorlab Thorlabs Inc., Newton, NJ, USA). This mechanism was modified in-house to be driven by a servo-controlled DC motor (RE-35, 90 W, Maxon motor AG, Sachseln, Switzerland) via a precision lead-screw. Such an arrangement provided vibration-free motion and very large mechanical stiffness, as well as the capacity to resist hundreds of newtons of perturbation. Thus, feedback control based on slaving the platform displacements on an externally applied force implemented an unconditionally stable control system of the admittance type.

### (c) Stimuli

The tactile stimulus was a lateral oscillation of the individual elements frequency-modulated by the temporal rate of change of the applied force, as illustrated in figure 1*b*. Let *M* be the coefficient of modulation of the stimulus oscillation frequency, *A* the amplitude, and *i* and *j* the rows and the columns of individual elements. The commanded positions were
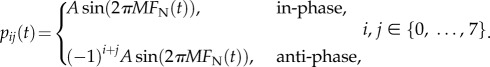


The stimulus was always produced with *A* = 0.1 mm, but only when *F*_N_ was greater than 0.5 N and smaller than an upper limit of 3.0, 6.0 or 9.0 N. The modulation coefficient *M* was set to either 4 or 12 N^−1^. The normal component, *F*_N_, of the measured force was processed by a first-order linear filter with a characteristic time of 50 ms prior to using it in the stimulus production.

### (d) Experiments and testing conditions

In experiment 1, the platform was immobile, and two conditions were tested. In one condition, all elements oscillated in phase. In the other condition, all pairs of adjacent elements oscillated in anti-phase. The in-phase condition tested two values of the coefficient of modulation, 4.0 and 12.0 N^−1^, and three values of the upper force limit, 3.0, 6.0 and 9.0 N. The control, anti-phase condition tested one value of the coefficient of modulation, 12.0 N^−1^, and two values of the upper force limit, 6.0, and 9.0 N. In experiment 1, observers were subsequently asked whether they were aware that the active surface did not move vertically.

In experiment 2, the experimental set-up was similar to that of experiment 1. The observers, however, maintained a steady force when pressing their finger on the stimulating surface which underwent no vertical displacement. Pre-recorded in-phase stimuli were applied to the fingertip during the steady-state plateau of the pressing force. The pre-recorded stimuli were acquired using the 12 N^−1^ modulation coefficient and the 6 N upper force limit, parameters that gave the strongest illusory percept and caused no fatigue.

Experiment 3 was similar to experiment 1 with the in-phase and anti-phase stimulation conditions, but the surface on which the observers pressed moved downward in proportion to the force applied, actually displacing their finger. The modulation coefficient was set to 12 N^−1^, and the motion stopped when the force was larger than the upper force limit set to 9 N. Because the psychophysical test method was the determination of points of subjective equivalence (PSE), the coefficient of proportionality between the force and the displacement varied such that the total displacement of the platform was in the range from 2 to 15 mm. The displacement of the surface occurred only for monotonically increasing pressing force. In the rare cases during a trial when the force was not monotonically increasing to its upper limit, the trial was discarded and a new one initiated.

### (e) Procedures

The observers sat comfortably with their right arm resting on a cushioned block that they held with their fingers, except the index finger. The stimulator was set behind the armrest and its height was individually adjusted, so that when resting the index finger on it, the distal phalange was sloping down at an angle of approximately 60° from the horizontal. In experiments 1 and 3, the lights in the experimental room were dimmed, and the observers wore a blindfold and sound-attenuating headphones. They received no feedback. In experiment 2, the lights were dimmed, and the observers watched force readings displayed on a screen to monitor their motor output.

In experiment 1, no familiarization nor pre-testing phase was needed. For each trial, the observers were instructed to put their index finger on the active surface and to push on it at a rate of their choosing until it stopped producing tactile stimulation (that is, when the upper force limit was reached). Then, they were to release their push steadily until the stimulation stopped (that is, when the 0.5 N lower force limit was reached again). At this point, they were to lift their finger from the surface and to report verbally the amount of perceived vertical displacement of their index finger in millimetres. The observers were explicitly told that they might experience no vertical displacement at all, and in this case they were to report 0 mm displacement. A typical force profile and time-course of the stimulus lasted about 1 or 2 s (figure 1*b*; electronic supplementary material, figure S2). The experiment comprised 18 randomized and balanced blocks of six trials for the in-phase condition (two values of the modulation coefficient and three levels of the upper force limit) and 12 trials for the anti-phase condition (two levels of the upper force limit). Each combination was repeated six times. The latter anti-phase conditions were mixed randomly among the trials of the in-phase condition. The entire experiment comprised 120 trials and took less than 20 min. The observers did not report any fatigue or decrease of their tactile sensitivity.

In experiment 2, the observers were aided by a visual feedback displayed on a computer screen helping them maintain a steady 6 N target force during which tactile stimulation was applied. Each observer repeated 100 trials, with 1.5 s pause after each trial. The entire experiment took approximately 10 min.

Experiment 3 used a two-alternative forced choice protocol to quantify more precisely the observers' perceived finger displacements. An experimental trial comprised a reference stimulus and a test stimulus presented in a random order. Upon a brief auditory cue, observers pressed on the stimulator and raised the finger once the platform stopped moving. A second auditory cue prompted the observer to repeat the same procedure and to report which stimulus elicited the greatest amount of perceived finger displacement. The reference stimulus always corresponded to an actual displacement of 5 mm combined with tactile stimulation of one of the two conditions. The test stimuli had seven different displacement magnitudes, which were based on the results of pre-testing trials and had no tactile stimulation. Each stimulus was presented 10 times together with the reference, leading to 140 randomized trials grouped in two blocks separated by rest periods. Two pre-testing blocks of trials with test stimuli of 2, 5, 10 and 15 mm displacement, each of which was presented four times, resulted in 32 trials presented in a randomized order. The smallest and largest vertical displacements were selected such that the observers could report with certainty whether the reference displacement was smaller or larger than the test for both in-phase and anti-phase conditions. These values were typically 2 and 10 mm. Seven uniformly spaced test stimuli were then selected. The overall experiment took less than 60 min.

### (f) Motor behaviour analysis

The effect of motor behaviour on perceived surface displacement was analysed from the results of experiment 1. To this end, the finger pressing force was recorded at an 80 Hz sampling rate. The trial durations were measured from the instant the finger pressing force first crossed the 0.5 N lower force limit to the instant when it dropped back to 0.5 N. The numbers of trials were different for the two conditions, so the median values of the trial durations among trials corresponding to the same condition were computed. The non-parametric Friedman test was applied to the resulting data (‘friedman.test’ function of R statistical software).

### (g) Testing methods and data analysis

In experiment 1, immediately after each trial, the observers reported verbally in millimetres any perceived vertical displacement of their fingertip. The observers' verbal reports were recorded by the experimenter. Prior to the experiment, the observers inspected visually and haptically a vernier calliper which was opened at 1.0, 5.0 and 10.0 mm in order to help them calibrate their subjective estimates. The Durbin test (non-parametric, balanced incomplete block design) was used to estimate the significance of the effect of the coefficient of modulation and of the upper force limit on the perceived displacement. For the control condition, the test was only used to assess the effect of the variation of the upper force limit. For the main condition, the test was used to assess the effect of the upper force limit variation for a fixed coefficient of modulation. To assess the effect of the variation of the coefficient of modulation, the test was applied to the combined data for all upper force limits. Calculations were performed using the function ‘durbin.test’ of R statistical software (package ‘agricolae’).

In experiment 2, observers simply reported whether or not they felt their finger move.

In experiment 3, the responses of each subject in the experiment block were fitted with probit models, one for in-phase and one for anti-phase condition for every subject. The PSEs were estimated to be the values of test stimuli for which the model gave 0.5 probability for the test and reference conditions. The perceptual biases were computed by subtracting the reference displacement from the PSEs. The significance of the biases (compared with zero) and the effect of the stimulation type on the bias were analysed using Wilcoxon ranked-sum test (‘wilcoxon.test’ of R statistical software). The magnitudes of biases were also compared with the median values of the perceived displacements as measured in the first experiment.

### (h) Estimation of mechanical energy exchange

Mechanical work was assumed to be a quantity to which different tactile receptors are tuned to respond [22]. In the in-phase condition, during a half-period of oscillation, the skin was laterally entrained by an amount of *δ* = 0.1 mm, and then brought back to rest as illustrated in electronic supplementary material, figure S1. An underestimate of the mechanical work spent in the skin and recovered from it can be obtained from the evaluation of the change in elastic energy in the finger. The main source of change of elastic energy in the finger came from the bulk displacement of the skin surface relative to that of subcutaneous tissues. The bulk elasticity of the human fingertip is typically of the order *k_b_* = 1.0 N mm^−1^ [23]; therefore, the elastic energy spent during the loading phase was *W_b_* = 0.5*k_b_δ*^2^. During one period, about 2*W_b_* = 1.0 × 10^−5^ J was exchanged between the stimulator and the finger. In the anti-phase condition, the finger bulk displacement was negligible. The change of elastic energy resulted mostly from locally stretching and compressing skin patches between neighbouring surfaces. There were about 50 patches of skin, 0.5 × 1.2 mm in size, that were stretched and relaxed during a half-period of oscillation by an amount of 2*δ* = 0.2 mm. Coincidentally, the local stiffness of such patches of skin deformed under differential tangential traction is also of the order *k_l_* = 1.0 N mm^−1^ [24]. Therefore, in the anti-phase condition, during one period of oscillation, the mechanical energy exchanged between the stimulator and the finger was about 2*W_l_* = 2[50 × 0.5*k_l_*(2*δ*)^2^], which is 2.0 × 10^−3^ J; that is, *W_l_* ≈ 200 *W_b_*.

### (i) Compared mechanical responses of granular material and stimulus

**Figure 2.**
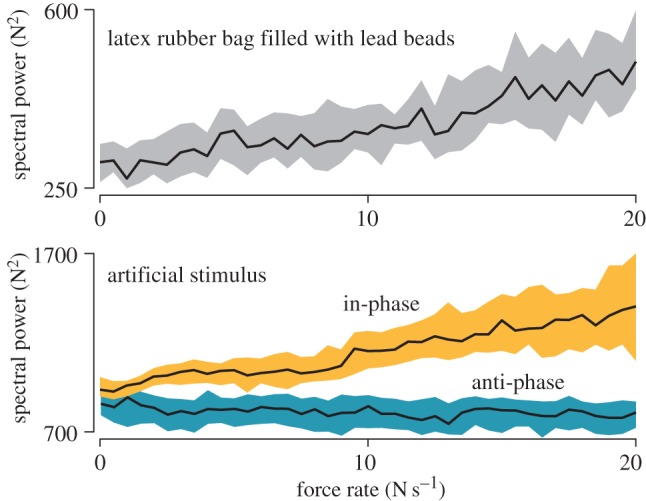
Spectral power of the in-phase stimulus and of force fluctuations elicited when pressing on granular material. The granular material, represented by a latex rubber bag filled with beads, was pressed by the experimenter. The in-phase and anti-phase stimuli (figure 1) were produced by the stimulation device impinging on a block of silicon rubber, simulating a finger. The signal power in the 200–1000 Hz frequency band were plotted against the rate of normal force change. The solid lines denote median values, the shaded regions give non-parametric estimates of their 95% CIs. (Online version in colour.)

The spectral properties of cutaneous stimulation induced by a change of pressing force were compared with the spectral properties of the mechanical response of granular material to a similar changing force. A 1.5 mm thick bag made of latex rubber filled with 2 mm lead shot was used as a model granular material. The bag was set on a rigid plate, forming a contact of an area of approximately 15–20 cm^2^. The plate was connected to high-resolution piezoelectric force transducers (tangential direction: model 9217A and charge amplifier 5018; normal direction: model 9313AA1 and charge amplifier 5073; Kistler Instrumente AG) through an arrangement that structurally separated the force components by a factor greater than 1 : 1000. The normal and tangential force component measurements were sampled at 10 kHz. The experimenter applied a cyclic force to the bag at 1–2 Hz with a magnitude reaching 10 N. The power spectrum in the band 200–1000 Hz was computed using the fast Fourier transform applied to 128 sample bins of the tangential force signal and then related to the rate of change of the normal force component. The same procedure was used to analyse the stimulus delivered by the tactile stimulator. The experimenter pressed on the stimulation device (figure 1*a*) turned upside down and brought into contact with a small block of silicon glued to the sensor plate. The device was programmed to produce the in-phase or anti-phase stimulus with the coefficient of modulation set to 4 N^−1^ and no upper force limit. Figure 2 shows the median values of the spectral power of the frequency components and their 95% CIs (estimated using non-parametric Wilcoxon statistics) determined as a function of the rate of change of the normal force filtered with 10 Hz lowpass filter. The curves were estimated from 117 loading–unloading cycles of the bag, 130 loading–unloading cycles for in-phase stimulation and 104 loading–unloading cycles for anti-phase stimulation. The graphs clearly highlight the similarity of the in-phase stimulus and the dissimilarity of the anti-phase stimulus with the natural response of a granular material.

## 3. Results

From the subjective reports collected, seven of ten observers participating in experiment 1 were certain that the surface was actually moving vertically and expressed disbelief after the experiment when the experimenter informed them that the surface was rigidly connected to the table. Three observers suspected that the surface was stationary, but still vividly felt a displacement of their finger. The magnitudes of apparent finger displacement reported by subjects are provided in the electronic supplementary material.

### (a) Stimulus specificity

**Figure 3.**
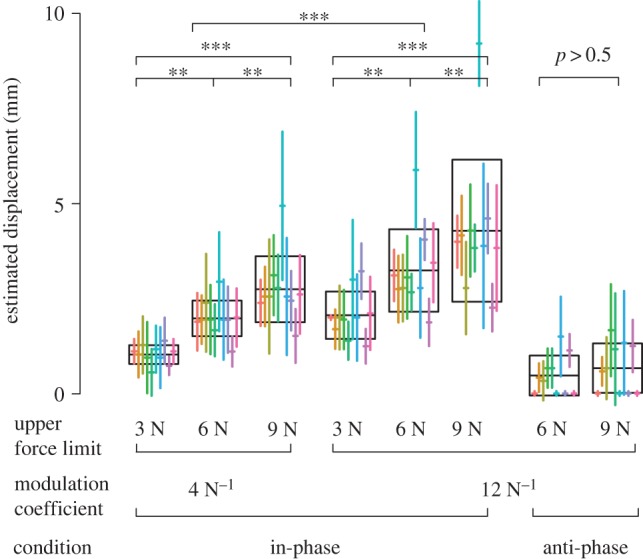
Results of experiment 1 for *n* = 10 observers. Crossed vertical lines denote the average and standard deviation (s.d.) for each individual participant. The data are plotted against the upper force limit and the coefficient of modulation. The group average and s.d. are shown with boxes. The effect of the upper force limit and of the modulation coefficient was tested with the non-parametric, conservative Durbin test. The effect of the upper force limit on perceived displacement was significant only for the in-phase stimulus.

In the in-phase condition of experiment 1, the perceived finger displacement, which reached up to 5 mm, scaled linearly with the upper force limit of the applied force (see figure 3). Yet, the upper force limit was not the sole factor in the perceived displacement. The coefficient of modulation of the oscillation frequency substantially influenced the perception of finger displacement. In contrast, in the anti-phase stimulus, the upper force limit had almost no effect on perceived displacement. Four observers consistently reported no vertical displacement at all in the case of anti-phase skin oscillations. In total, observers reported no displacement in 56% of the trials for the anti-phase condition, compared with reports of no displacement in only 3% of the trials for the in-phase condition.

No observer participating in experiment 2 reported any significant perceived finger displacement. One of them did report that occasionally a fleeting sensation of displacement was experienced, but it was very weak, ‘much smaller than one millimetre’ as the observer phrased it.

**Figure 4.**
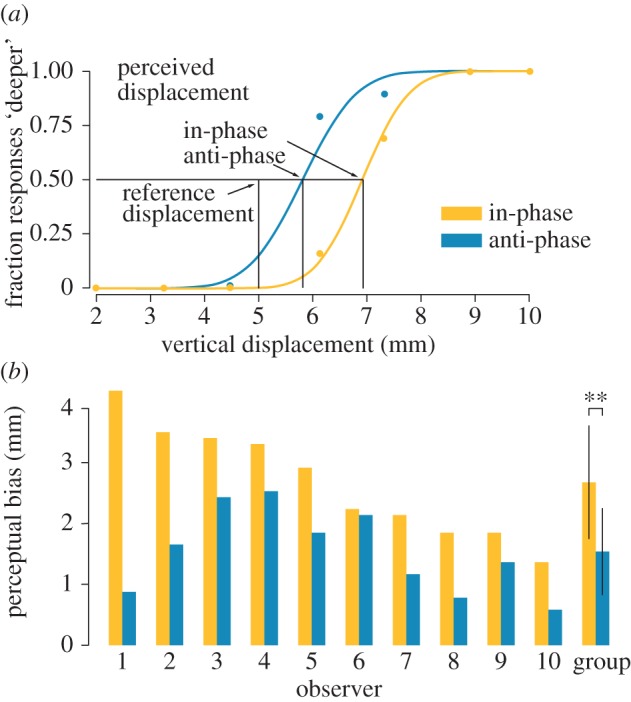
Results of experiment 3. (*a*) Psychometric curves of a representative observer with a 5 mm reference displacement of the surface. The point at which the curve ordinates are equal to 0.5 corresponds to the same perceived displacement of the platform with and without skin stimulation. (*b*) The points of subjective equivalence (same perceived displacement) for *n* = 10 observers with group average and standard deviation. (Online version in colour.)

In experiment 3, when the displacement of the active surface in response to the pressing force was accompanied by in-phase stimulation of the skin, the observers perceived significantly larger displacement of the surface (*p* < 0.01, *V* = 55) than when no stimulation of the skin was applied. Surprisingly, anti-phase stimulation also increased for some observers the amount of perceived finger displacement (*p* < 0.01, *V* = 55), but the effect was much smaller than for in-phase stimulation (*p* < 0.01, *V* = 55; see figure 4*b*). The biases in the in-phase condition were slightly smaller than verbally reported finger displacements; this result was statistically significant, but not strong (*p* < 0.05, *W* = 21). This was in contrast with the anti-phase condition, where the biases were significantly greater than those reported verbally (*p* < 0.001, *W* = 99).

### (b) Absence of effect on motor behaviour

Electronic supplementary material, figure S2 shows the raw data from experiment 1 for a typical observer for an upper force limit of 6.0 N and a modulation coefficient of 12.0 N^−1^. The trial durations did not vary significantly between the two conditions (Friedman test, *p* > 0.3; average and standard deviation, 1.82 ± 0.55 s for the in-phase condition and 1.84 ± 0.52 s for the anti-phase condition), indicating that the observers responded similarly to the cessations of the stimulus past the upper and lower limits of the pressing force.

## 4. Discussion

### (a) Factors influencing the illusory sensation of movement

The correlation of an oscillatory stimulation of the fingertip skin with the rate of change of the pressing force elicited an illusory sensation of displacement of the finger, as reported by the ten observers participating in the study. It was important to ensure that the observers did experience an illusory sensation and did not simply base their judgements on the external cues related to the experimental protocol. A possible strategy would have been to guess that a higher upper force limit corresponded to larger displacements. However, the same strategy could have been employed in the anti-phase condition. Moreover, they consistently reported larger displacements for the higher values of the modulation coefficient, which were unrelated to the upper force limit. Another strategy could have been to estimate the number of stimulus oscillations to associate this estimate with a displacement. Tripling this number, however, did not triple the displacement estimates. More importantly, there is no reason why the observers would decide that the anti-phase stimulus should correspond to no displacement, unless it indeed produced no such percept. We can conclude that subjects actually experienced an illusory displacement of their finger and did not rely on possible cognitive strategies using external sources of information. Experiment 3, employing a moving active surface, provided an objective quantification of the illusion. In-phase stimulation of the fingertip simultaneously with platform displacement increased the subjective perception of the finger displacement in all subjects. This effect was somewhat smaller than in the main task, which can partially be explained by differences in the testing conditions. In particular, owing to the experimental necessity, observers received no tactile stimulation during the release phase of a trial.

### (b) Possible mechanisms that can be excluded

The illusory sensation of displacement could have a purely cutaneous origin. For example, it could arise from the properties of slowly adapting cutaneous receptors which have been demonstrated to respond vigorously to smooth isotonic finger movement [25]. The specific excitation of cutaneous receptors created by the stimulation could explain the occurrence of a sensation of finger movement [4,26,27]. The results of experiment 2 showed unequivocally that cutaneous stimulation alone is insufficient to elicit a percept of finger displacement.

The sensation of illusory movement could originate in the properties of muscular, articular and tendinous receptors. For instance, the periodic stimulation of muscle spindles by means of superficial vibration can create an illusion of limb movement while the limb remains immobile [28]. This illusion has a characteristic time of about 5 s and would not develop within the short trial durations in this study. Moreover, we see no reason why the illusion would fail to develop in the anti-phase condition or why it would require a force-modulated input. Force production itself can alter the perception of limb position [29,30]. However, it is unlikely to be the reason for the percept, which, for the same upper force limit, depends on the spatial pattern of stimulation and on the modulation coefficient.

### (c) Likely mechanisms

It is thus difficult to ascribe the origin of the described effect to a single sensory modality. It may instead result from the integration of tactile information with the information about the force applied to the surface, possibly with the contribution of an efference copy of the motor commands. But why would the specific force rate-modulated skin oscillations elicit a percept of limb displacement?

A possible explanation is that the particular law of stimulation used here resembles the sensory congruency typical of interaction with aggregate materials, such as sand, snow and others. In such materials, slow compacting is associated with discrete impulses of energy release at a rate that is monotonously related to the rate of deformation of the bulk [31,32], which in turn increases with the rate of change of the applied force. These sensed impulses of energy release can be used by the brain to estimate the bulk displacement. Empirical measurements demonstrated the similarity between the spectral properties of the fluctuations in reaction accompanying the deformation of granular objects and of the oscillations of the finger skin induced by the artificial stimulus.

The dependency of the illusory percept on the upper force limit and modulation coefficient can be explained by assuming that a touched object responds with a fixed number of force pulses per unit length of its deformation or displacement. In this case, a greater force applied by the finger for the same modulation coefficient corresponds to a greater number of force pulses and hence to greater finger displacement. Similarly, a greater modulation coefficient results in a greater number of force pulses for reaching the same value of applied force, which again corresponds to greater finger displacement. These effects are apparent in the judgements of all observers.

A possible mechanism responsible for the illusion would thus appeal to previously learned sensorimotor association that relates the change of the force applied to an object and the frequency of occurrence of microscopic mechanical events accompanying object's deformation. This mechanism can be related to the notion of an ‘internal model’—a neural representation of pre-learned sensorimotor relationships associated with one's body actions [33], and with manipulating the external objects [34]. There is previous evidence that the processing of somatosensory inputs with the internal models may take place in the neocortex or in the cerebellum [33,35].

### (d) Specificity of in-phase oscillations

Such reasoning, however, does not explain the difference in the percepts elicited by in-phase and anti-phase stimulation conditions. Several factors can be invoked. It can be hypothesized that the dynamic strain fields induced in the skin by the anti-phase stimulus underwent destructive interferences taking place selectively according to the distance from the skin surface. Neural factors could also have been at play, for instance owing to inhibitory mechanisms that are thought to take place at multiple levels of the somatosensory pathway [19,20,36–38].

The magnitude of mechanical energy exchanged between the active surface and the fingertip cannot explain the difference of effects of the in-phase and anti-phase stimuli. The excitation of cutaneous mechanoreceptors can be related to local mechanical quantities, such as strain energy [22]. In our experiment, the amount of mechanical energy exchanged with the skin during one oscillation of the anti-phase stimulus was 100-fold greater than that exchanged during one oscillation of the in-phase stimulus, implying that the percept should be dramatically stronger in the anti-phase condition. The absence of such effect suggests a weak link between the sheer magnitude of a tactile stimulus and the sensations that it elicits.

Interestingly, the results of experiment 3 indicated that the perception of at least some of the observers was significantly biased by anti-phase skin stimulation, albeit to a much smaller degree than by the in-phase stimulation. These results contrast with those of experiment 1, where most subjects experienced no illusory displacement with anti-phase stimulation. The main difference between these two cases was a complete absence of actual movement in the conditions of experiment 1 and a cue combination situation in the conditions of experiment 3. The difference in perceptual outcomes is compatible with the ‘cue promotion’ hypothesis described in the visual modality [39]. It is hypothesized that different perceptual cues referring to a single quantity interact with each other before their combination takes place. In experiment 1, a single cue was available to the observers; thus it was the sole contributor to the sensation of movement, explaining the large difference between the in-phase and the anti-phase condition. In experiment 3, two cues were available simultaneously and thus could interfere prior to being fused.

## 5. Conclusion

### (a) Practical applications

The subjective experience elicited by the stimulus that we have described bears an uncanny resemblance to the sensation experienced when pressing a push button. It requires only a frequency-controlled vibration generator and a load sensor to be realized. This simplicity is highly attractive in human–machine interfaces, such as in flat tactile input panels, levers, steering wheels and so on. In the surgical domain, the artificially magnified vibrations of a hand-held instrument in response to its interaction with soft tissues were shown to significantly enhance tissue anomaly detection performance [40]. These results can be re-examined in the light of the present findings, because magnified vibratory inputs are likely to also elicit magnified sensations of tool movement. Likewise, the recent application of similar techniques to the enhancement of robotic surgery user interfaces could yield similar benefits [41]. Conversely, in performance-critical user interfaces such as in vehicle cockpits or telesurgical workstations, certain uncontrolled mechanical oscillations could accidentally induce illusory sensations of movement, leading to grave and undesirable consequences.

### (b) Theoretical implications

We have provided support to the notion that proprioception, traditionally associated with muscle, tendon, joint and skin receptors [42,43], may integrate prior information about the mechanical consequences of interacting with external objects, which in this case are in the form of microscopic fluctuations caused by object deformation. In this respect, proprioception is similar to other sensory modalities where the essential role of prior statistical information about the world in the formation of percepts is commonly accepted. Our results suggest a counterintuitive interpretation of the source of sensory experience when interacting with external objects. When pressing on a movable object such as a push button, the brain does not determine the displacement of the button solely from the displacement of the finger, as measured by the joint, tendon and muscles receptors. Instead, the brain uses prior knowledge of the button's microscopic oscillations properties, which, when correlated with force estimates, contribute to a sensation of displacement of the finger.

## Supplementary Material

Figure S1.

## Supplementary Material

Figure S2.

## Supplementary Material

Experimental data
