# Supplementary figures and images for: The brain uses extrasomatic information to estimate limb displacement
Source: Proc Biol Sci. 2015 Sep 7;282(1814):20151661. doi: 10.1098/rspb.2015.1661 (PMC4571714; doi:10.1098/rspb.2015.1661)

0.1 mm  $\rightarrow$

in-phase

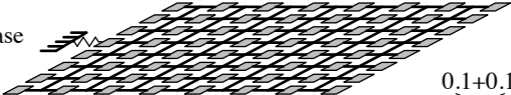

0.1+0.1 mm

anti-phase

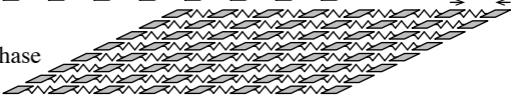

Supplement: Figure S1. [file rspb20151661supp1.pdf]

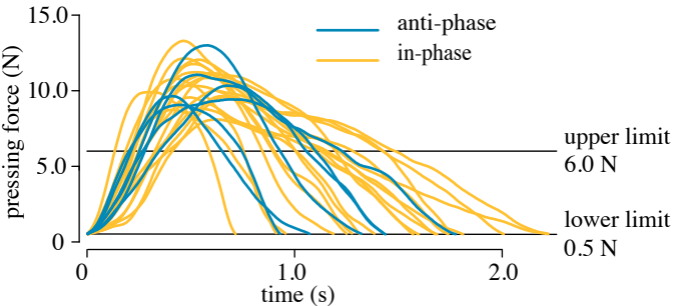

Supplement: Figure S2. [file rspb20151661supp2.pdf]
